# Evolutionary Diversification of Alanine Transaminases in Yeast: Catabolic Specialization and Biosynthetic Redundancy

**DOI:** 10.3389/fmicb.2017.01150

**Published:** 2017-06-26

**Authors:** Ximena Escalera-Fanjul, Carlos Campero-Basaldua, Maritrini Colón, James González, Dariel Márquez, Alicia González

**Affiliations:** Instituto de Fisiología Celular, Departamento de Bioquímica y Biología Estructural, Universidad Nacional Autónoma de MéxicoMexico City, Mexico

**Keywords:** orthologous genes, alanine metabolism, functional diversification, alanine transaminase, transcriptional regulation

## Abstract

Gene duplication is one of the major evolutionary mechanisms providing raw material for the generation of genes with new or modified functions. The yeast *Saccharomyces cerevisiae* originated after an allopolyploidization event, which involved mating between two different ancestral yeast species. *ScALT1* and *ScALT2* codify proteins with 65% identity, which were proposed to be paralogous alanine transaminases. Further analysis of their physiological role showed that while *ScALT1* encodes an alanine transaminase which constitutes the main pathway for alanine biosynthesis and the sole pathway for alanine catabolism, *Sc*Alt2 does not display alanine transaminase activity and is not involved in alanine metabolism. Moreover, phylogenetic studies have suggested that *ScALT1* and *ScALT2* come from each one of the two parental strains which gave rise to the ancestral hybrid. The present work has been aimed to the understanding of the properties of the ancestral type *Lacchancea kluyveri LkALT1* and *Kluyveromyces lactis KlALT1*, alanine transaminases in order to better understand the *ScALT1* and *ScALT2* evolutionary history. These ancestral -type species were chosen since they harbor *ALT1* genes, which are related to *ScALT2.* Presented results show that, although *LkALT1* and *KlALT1* constitute *ScALT1* orthologous genes, encoding alanine transaminases, both yeasts display *Lk*Alt1 and *Kl*Alt1 independent alanine transaminase activity and additional unidentified alanine biosynthetic and catabolic pathway(s). Furthermore, phenotypic analysis of null mutants uncovered the fact that *Kl*Alt1 and *Lk*Alt1 have an additional role, not related to alanine metabolism but is necessary to achieve wild type growth rate. Our study shows that the ancestral alanine transaminase function has been retained by the *ScALT1* encoded enzyme, which has specialized its catabolic character, while losing the alanine independent role observed in the ancestral type enzymes. The fact that *Sc*Alt2 conserves 64% identity with *Lk*Alt1 and 66% with *Kl*Alt1, suggests that *Sc*Alt2 diversified after the ancestral hybrid was formed. *ScALT2* functional diversification resulted in loss of both alanine transaminase activity and the additional alanine-independent *Lk*Alt1 function, since *ScALT2* did not complement the *Lkalt1Δ* phenotype. It can be concluded that *LkALT1* and *KlLALT1* functional role as alanine transaminases was delegated to *ScALT1*, while *ScALT2* lost this role during diversification.

## Introduction

Gene duplication plays a central role in evolution, enhancing organism robustness, and providing material for the emergence of new or specialized functions. Different models have been posed to explain which is the possible fate of duplicated genes. The *gene-dosage amplification* model considers that gene doubling is not followed by functional diversification and that after duplication both gene copies are retained, augmenting the ancestral function, which could represent a selective advantage (Kondrashov et al., 2002; Kondrashov and Kondrashov, 2006; Tang and Amon, 2013). Conversely, three models including functional divergence have been developed; *loss of function* model sustains that as a result of the accumulation of loss-of-function mutations one gene copy is lost, becoming a pseudogene (Conrad and Antonarakis, 2007). Alternatively, the *neofunctionalization* model considers that one of the copies conserves the ancestral function, while the other one acquires a new adaptative function by the accumulation of neutral mutations (Ohno, 1970). And finally, the *subfunctionalization* model proposes that the accumulation of degenerative mutations split or specialize the ancestral function(s) between the two paralogous, therefore both copies are necessary to perform the function which in the ancestor was carried out by a single gene (Hughes, 1994; Innan and Kondrashov, 2010; Sikosek et al., 2012; Espinosa-Cantú et al., 2015).

**FIGURE 1:**
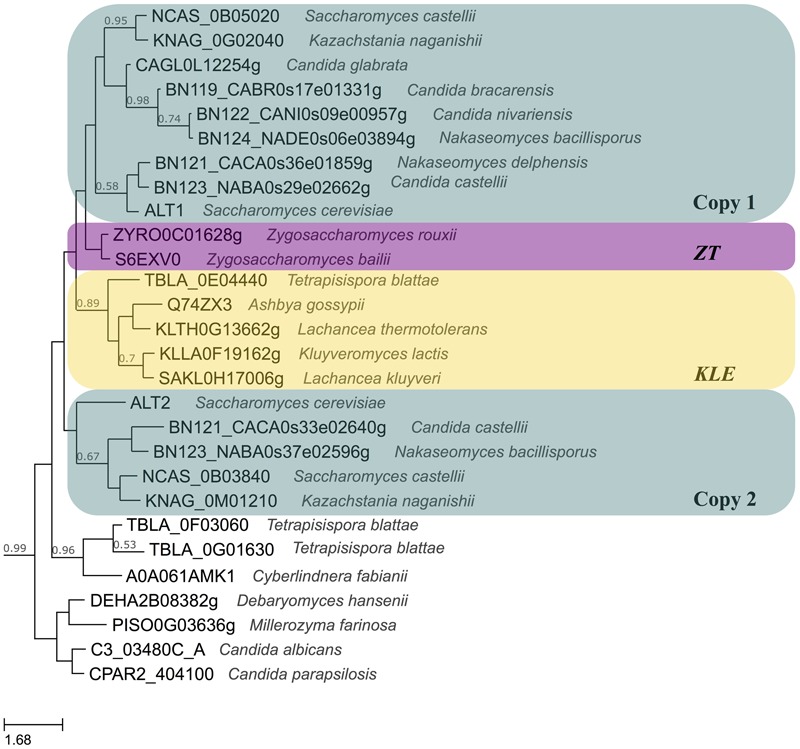
Evolutionary relationships of Alts from yeasts. The phylogeny was modified from http://phylomedb.org alt2 tree in phylome 410. URL: http://phylomedb.org/?q=search_tree&seqid=alt2

For many years, it was accepted that the *Saccharomyces cerevisiae* (*S. cerevisiae)* lineage arose from a Whole Genome Duplication (WGD), making this yeast an interesting model to study diversification of paralogous genes (Wolfe and Shields, 1997; Kellis et al., 2004). Recently, a phylogenetic study found compelling evidence indicating that *S. cerevisiae* lineage arose from an interspecies hybridization between one strain related to the *Kluyveromyces, Lachancea* and *Eremothecium* (*KLE*) clade and another one related to *Zygosaccharomyces rouxii* and *Torulaspora delbrueckii* (*ZT*). Although whether the hybrid was the result of the fusion of two diploid cells or two haploid cells that underwent a WGD, is still an open question, both scenarios result in the formation of an allotetraploid with two copies of every gene. After the allotetraploid was formed, intragenic recombinations, full gene conversion, differential gene loss and selection pressures shaped *S. cerevisiae* genome to the one we observe today, harboring conserved blocks of duplicated genes (Marcet-Houben and Gabaldón, 2015). Thus, the paralogous genes that are observed today can both come from one of the species involved in the formation of the hybrid, or each paralogue could have originated from each one of the parental species which constituted the hybrid. In *S. cerevisiae ScALT1* and *ScALT2* genes are part of the duplicated blocks coming from the hybridization; however, while *ScALT1* comes from the *ZT* parental strain, *ScALT2* parental is closer to the *KLE* clade, as it can be seen in **Figure 1** (Huerta-Cepas et al., 2014).

*ScALT1* and *ScALT2* are annotated as putative alanine transaminases, however, previous studies of alanine metabolism in *S. cerevisiae* and *Sc*Alt1 and *Sc*Alt2 physiological roles, showed that when this yeast is grown on glucose and ammonium sulfate as carbon and nitrogen sources, (biosynthetic conditions), alanine is mainly synthesized by the *ScALT1*-encoded alanine transaminase, while the *ScALT2-*encoded protein does not contribute to alanine biosynthesis (Peñalosa-Ruiz et al., 2012). Furthermore, *Scalt1Δ* mutants are not alanine auxotrophs, indicating the existence of a hitherto undescribed alanine biosynthetic pathway, that could be constituted by the previously described glutamine transaminase, which has been proposed to give rise to alanine and α-ketoglutaramate, since it uses pyruvate and glutamine as preferred substrates (Soberón and González, 1987), providing an alternative alanine biosynthetic pathway and supporting the observation of a wild type growth phenotype of the *Scalt1Δ* mutant. Worth mentioning is the fact that the glutamine transaminase could not provide an alanine catabolic pathway, since α-ketoglutaramate is spontaneously cyclized and used by the ω-amidase to produce α-ketoglutarate (Soberón and González, 1987). Accordingly, it was found that the *Scalt1Δ* mutant strain does not grow in the presence of alanine as sole nitrogen source, demonstrating that the *Sc*Alt1 protein constitutes the only pathway for alanine catabolism, and that *Sc*Alt2 does not play a role in either alanine biosynthesis or catabolism. In addition, it was found that *Sc*Alt2 was devoid of alanine transaminase activity, and consequently, *Sc*Alt2 function remains to be discovered (Peñalosa-Ruiz et al., 2012).

The physiological roles and the evolutionary histories of *ScALT1* and *ScALT2* are appealing, since these genes are inferred to descend from each of the different parental strains involved in the formation of the hybrid that gave rise to the *S. cerevisiae* lineage, and consequently, it is not possible to tell if at the moment of the hybridization, these two genes, *ScALT1* and *ScALT2*, had the same function or had already functionally diverged. Worth of mention is the fact that, we have previously shown that *Sc*Alt2 does not bear alanine transaminase activity and it might have acquired by either *subfunctionalization* or *neofunctionalization* a distinct physiological role. To better understand how *ScALT1* and *ScALT2* diverged, and the putative ancestral function of *ScALT2* we decided to study the functional role of presumed alanine aminotransferases from pre-WGD yeasts that share a closest ancestor with the parental lineage which carried *ScALT2*. As such this could be regarded as a proxi to “ancestral type” *ScALT2*. For this, *Lachancea kluyveri* (*LkALT1*) and *Kluyveromices lactis* (*KlALT1*), were selected for functional characterization of their encoded enzymes. *LkALT1* and *KlALT1* were identified by sequence identity, synteny and phylogenetic analysis as the sole orthologous genes of both *ScALT1* and *ScALT2* present in *L. kluyveri* and *K. lactis*, respectively. Both species belong to the so called *KLE* clade, which is the closest one to *ScALT2* parental strain, and share a more distant common ancestor with the *ZT* clade, from which *ScALT1* was originated (Marcet-Houben and Gabaldón, 2015).

Results presented in this paper indicate: (i) *LkALT1* and *KlALT1* are functionally equivalent to *ScALT1* functional orthologous genes, encoding alanine transaminase with a characteristic Ping Pong kinetic mechanism, (ii) while for *S. cerevisiae* alanine catabolism is strictly ScAlt1-dependent, in *L. kluyveri* and *K. lactis* additional unidentified pathways contribute to both alanine biosynthesis and catabolism, (iii) *Lkalt1Δ* and *Klalt1Δ* mutants retain alanine transaminase activity indicating the existence of a second alanine transaminase encoding gene in *L. kluyveri* and *K. lactis*, (iv) *Lk*Alt1 and *Kl*Alt1 enzymatic activity profile, respectively, follow *LkALT1*and *KlALT1* expression profile, and v) that as shown by reciprocal complementation tests, *Lk*Alt1 and *Kl*Alt1 have a yet unknown alanine-independent alternative function which neither *ScALT1* nor *ScALT2* can complement.

## Materials and Methods

### Strains

**Table 1** describes the characteristics of the strains used in the present work. All strains constructed for this study were derivatives of *CLA1-2* (*ura3 leu2::LEU2*), *Lk156-1* (*ura3*) or *Kl155* (*ade2 his3 ura3*) for *S. cerevisiae, L. kluyveri*, and *K. lactis*, respectively. Mutant strains as *Scalt1Δ* (*CLA1-2-1*), *Scalt2Δ* (*CLA1-2-2*), *Scalt1Δ Scalt2Δ* (*CLA1-2-D*) (Quezada et al., 2008; García-Campusano, 2009). The *L. kluyveri Lkalt1Δ* (*Lk156-2*) mutant strain was kindly provided by Dr. Lina Riego, and was obtained by replacing the ORF of *LkALT1* with the selectable marker *kanMx4*. The *LkALT1* gene was replaced by homologous recombination using a module containing the *kanMX4* cassette (1469 bp) flanked by 1146 bp of 5′UTR (-1181 to -34) and 1207 bp of 3′UTR (+1604 to +2811) sequences of *LkALT1*. This module (3608 bp) was amplified by overlapped extension PCR with deoxyoligonucleotides X7 and X8 (-1007 to +2061) using a template built up by three independent modules: (i) the *LkALT1* 5′UTR amplified using the X1 and X2 deoxyoligonucleotides and genomic DNA from strain *Lk156-1* as a template, (ii) the *kanMX4* module which was amplified from the pFA6a plasmid using deoxyoligonucleotides X3 and X4, and (iii) the *LkALT1* 3′UTR amplified using deoxyoligonucleotides X5 and X6 and genomic DNA from strain *Lk156-1* as a template. The PCR product was transformed into the *Lk156-1* strain. Transformants were selected for G418 resistance (200 μg ml^-1^). Deoxyoligonucleotides X9/X10 and X11/X12 were used to verify the construction *Lkalt1*Δ*::kanMX4*, 1547 and 1851 bp (from -1345 of the *KlALT1* 5′UTR to +216 of *kanMX4* and from +874 of *kanMX4* to +2879 of 3′UTR of *KlALT1*), respectively. The deoxyoligonucleotides sequences are indicated in Supplementary Table S1.

**Table 1:** *Saccharomyces cerevisiae, Lacchancea kluyveri*, and *Kluyveromyces lactis* strains used in this work.

Strain	Relevant genotype	Reference
*CLA1-2*	*MATα ScALT1 ScALT2 ura3 leu2::LEU2*	Quezada et al., 2008
*CLA1-2-1*	*MATa Scalt1Δ::kanMX4 ScALT2 ura3 leu2::LEU2*	García-Campusano, 2009
*CLA1-2-2*	*MATa ScALT1 Scalt2Δ::kanMX4 ura3 leu2::LEU2*	García-Campusano, 2009
*CLA1-2D*	*MATa Scalt1Δ::kanMX4 Scalt2Δ::natMX4 ura3 leu2::LEU2*	García-Campusano, 2009
*LK 156-1*	*Mata LkALT1 ura3*	Montalvo-Arredondo et al., 2015
*Lk Y156-2*	*MATα Lkalt1Δ::kanMX4 ura3*	This study
*Kl 155*	*MATα ade2 his3 ura3*	Colón et al., 2011
*Kl 155-2*	*MATα Klalt1Δ::kanMX4 ade2 his3 ura3*	This study

The *K. lactis Klalt1Δ* (*Kl155-2*) mutant strain was obtained by replacing the ORF of *KlALT1* with the selectable marker *kanMX4*. The *KlALT1* gene was replaced by homologous recombination using a module containing the *kanMX4* cassette (1507 bp) flanked by 987 bp of 5′UTR (-1169 to -222) and 1202 bp of 3′UTR (+1587 to +2771) sequences of *KlALT1*. This module (2495 bp) was amplified by overlapped extension PCR with deoxyoligonucleotides (-1169 to +2771) using a template built up by three independent modules: (i) the *KlALT1* 5’UTR amplified using the X13 and X14 deoxyoligonucleotides and genomic DNA from strain *Kl155* as a template, (ii) the *kanMX4* module which was amplified from the pFA6a plasmid using deoxyoligonucleotides X15 and X16, and (iii) the *KlALT1* 3’UTR amplified using deoxyoligonucleotides X17 and X18 and genomic DNA from strain *Kl155* as a template. The PCR product was transformed into the *Kl155* strain. Transformants were selected for G418 resistance (250 μg ml^-1^), the correct module integration was PCR confirmed using the genomic DNA of the G418-resistant transformants as a template and the deoxyoligonucleotides X19 in combination X20 and X21 in combination X22, these primers generated a module of 1495 and 1978 bp (from -1496 of the *KlALT1* 5′UTR to +216 of *kanMX4* and from +1106 of *kanMX4* to +3172 of 3′UTR of *KlALT1*), respectively. Deoxyoligonucleotides sequences are indicated in Supplementary Table S1.

### Growth Conditions

Strains were routinely grown on minimal medium (MM) containing yeast nitrogen base without amino acids and ammonium sulfate (Difco), glucose (2% w/v) was used as carbon source, and 40 mM ammonium sulfate or 7 mM alanine were used as the nitrogen source Amino acids needed to satisfy auxotrophic requirements were added at 0.01% (w/v). Culture growth was monitored by OD_600nm_ measurements at 30 m intervals using Bioscreen C (Oy Growth Curves Ab Ltd.) set at maximum shaking and 30°C. Otherwise, cells were incubated at 30°C with shaking (250 rpm).

### Plasmid Construction

Cloning into the pRS416 and YEpKD plasmids was performed as follows: to clone the *ScALT1* gene into pRS416 plasmid, a 2848 bp region between -931 bp upstream from the start codon and +137 bp downstream from the stop codon was amplified with deoxyoligonucleotides X23 and X24 using genomic DNA from the *S. cerevisiae* (CLA1-2) wild type strain as a template, after PCR amplification, and plasmid digestion with *Eco*RI and *Xho*I, cloning was carried out using the NEBuilder HiFi DNA Assembly Master Mix; to clone the *ScALT1* gene into YEpKD352 plasmid, a 2619 bp region between -937 bp upstream the start codon and +95 bp downstream the stop codon was amplified with deoxyoligonucleotides X25 and X26, also using genomic DNA from the CLA1-2 wild type strain as template; for the *ScALT2* gene, a 2626 bp region between -996 bp upstream from the start codon and +105 bp downstream from the stop codon was amplified with deoxyoligonucleotides X27 and X28 using genomic DNA from the *S. cerevisiae* (CLA1-2) WT strain as a template; for the *LkALT1* gene, a 2611 bp region between -920 bp upstream to the start codon and +281 bp downstream from the stop codon was amplified with deoxyoligonucleotides X29 and X30 using genomic DNA from the *L. kluyveri* (*Lk156-1*) wild type strain as a template; and for *KlALT1*, a 2619 bp region between -937 bp upstream from the start codon and +95 bp downstream to the stop codon was amplified with deoxyoligonucleotides X31 and X32 using genomic DNA from the *K. lactis* (*Kl155*) wild type strain as a template. The PCR products and pRS416 plasmid were digested with restriction enzymes and ligated after gel purification.

### Metabolite Extraction and Analysis

Cell extracts were prepared from exponentially growing cultures. Samples used for intracellular amino acid determination were deproteinized with perchloric acid. Intracellular alanine was determined by HPLC with an Ultrasphere ODS C18 column (Beckman Coulter) with ortho-phthalaldehyde (OPA) derivatization and a mobile phase consisting of a 30 min gradient from 10 to 75% methanol and 25 % 0.1 M potassium acetate buffer (pH 5.5) flowing at 1.5 ml min^-1^. Quantification was carried out with a fluorescent detector (Quezada et al., 2008).

### Cell Extract Preparation and Alanine Transaminase Enzymatic Assay

Yeast cells were harvested by centrifugation, and washed with cold water. Pellets were suspended in cold extraction buffer (50 mM HEPES, 1 mM PMSF, 1 mM EDTA, 1 mM DTT). For *Kl*Alt1 extraction buffer was supplemented with complete, mini protease inhibitor cocktail, by Roche. Cells were mechanically disrupted with glass beads. The resulting extract was centrifuged to eliminate cellular debris (5,000 rpm, 4°C, 15 min). The supernatant was recovered, and pyridoxal-5-phosphate was added to a 100 μM final concentration. Protein was measured by the method of Lowry (Lowry et al., 1951), using bovine serum albumin as a standard. Enzymatic assay was a modified version from (Graßl and Supp, 1985). The reaction mixture contained reaction buffer (pH7 100 mM Tris-HCl, 400 mM alanine, 24 mM α-ketoglutarate, 300 μM NADH, 40 μM pyridoxal-5-phosphate and 5 U/mL of lactate dehydrogenase). As control, assays were performed without alanine. To determine specific activity the slope obtained from this negative control was subtracted to obtained with the complete assay. All assays were performed at 340 nm, 25°C in a Varian Cary 50 spectrophotometer.

### Cloning and Expression

The *LkALT1* and *KlALT1* genes were PCR amplified using the deoxyoligonucleotides pairs X33/X34 and X35/X36, respectively. To amplify *LkALT1* genomic DNA of the *Lk156-1* WT strain was used as template. Alternatively, to amplify *KlALT1* genomic DNA of the *Kl155* wild type strain genomic DNA was used as template. Obtained PCR product and the pET-28a(+) plasmid were *Nhe*I/*Xho*I digested for *LkALT1* cloning and *Nde*I/*Xho*I digested for *KlALT1* cloning. After gel purification plasmids and inserts were ligated. Ligations were transformed into the DH5α *E. coli* strain. After plasmid purification, correct cloning was verified by sequencing. For heterologous expression, the Rosetta2 (DE3) *E. coli* strain was transformed. Selected clones were grown in LB medium supplemented with 50 μg ml^-1^ of kanamycin and 70 μg ml^-1^ 30 of chloramphenicol, cells were incubated at 37°C with shaking (250 rpm). When the cultures reached an OD_600__rmnm_ 0.75 the expression of the proteins was induced with 100 μmol/L of IPTG (Iso-Propil-Tio-Galactoside), incubated over night at 30°C with shaking (250 rpm). IPTG induced cells were harvested by centrifugation at 3750 rpm for 30 min.

### Rosetta 2(DE3) Whole Cell Soluble Protein Extract

Cells were thawed and resuspended in 4 ml of lysis buffer per 100 ml of culture (10 mmol/L imidazol, 50 mmol/L NaCl, and 10 mmo/L Tris-HCl pH8.0). Protein extracts were obtained by sonication (Ultrasonic Processor Model: VCX 130) with a tip sonicator keeping the tubes on ice; five cycles (60% amplitude, one second on and one second off for 1 min) with 1 min of incubation on ice between each cycle. After centrifugation at 17000 rpm for 10 min at 4°C, the supernatant was recovered, and pyridoxal-5-phosphate was added to a 100 μM final concentration.

### Affinity Chromatography

To purify alanine transaminase isozymes, supernatant obtained as stated above, was loaded on an equilibrated nickel column (Ni-NTA Agarose 100, Thermo Fisher Scientific), which was then washed 30 times with 30 mmol/L imidazol. The protein was eluted with 200 mmol/L imidazol and 100 μM final concentration of pyridoxal-5-phosphate was added. Protein homogeneity was verified by polyacrylamide gel electrophoresis 10% (SDS-PAGE) stained with Coomassie Blue (Supplementary Figure S1).

### Enzyme Kinetics and Analysis of Kinetic Data

Initial velocity measurements were performed by varying both substrates simultaneously (for *LkALT1* alanine from 1.00 to 20.00 mM and α-ketoglutarate from 0.05 to 1.50 mM; for *KlALT1* alanine from 4.00 to 80.00 mM and α-ketoglutarate from 0.25 to 5.00 mM), and the results where globally fitted to the equation for an enzyme with a ping-pong mechanism (Segel, 1975) with the Prism software GraphPad Prism 6.00 (Software Inc.).

### Northern Blot Analysis

Northern blot analysis was carried out as previously described by Struhl and Davis (1981). Total yeast RNA was prepared from 100 ml cultures grown to indicated OD_600__nm_ in MM with 2% glucose, ammonium sulfate, or alanine as a nitrogen source. PCR products were used as probes. For *LkALT1*, a 400 bp product was amplified with deoxyoligonucleotides X37 and X38; for *KlALT1*, a 897 bp PCR product was amplified with deoxyoligonucleotides X39 and X40; *Lk18s* and *Kl18s* were used as internal loading standards. Both *Lk18s* and *Kl18s* were amplified with deoxyoligonucleotides X41 and X42 a product of 476 bp and 477 bp was amplified for *LkALT1* and *KlALT1*, respectively.

### Nucleosome Scanning Assay (NuSA)

The nucleosome scanning assay was made to see the chromatin organization *LkALT1* and *KlALT1* promoter, and the procedure to the study of the positioning of nucleosomes on promoters was made as described by Infante et al. (2011). When the cultures reached an OD_600__nm_ of 0.6 at, genetic DNA was obtained of *Kl155*, and *Lk156-1* strains grown on MM with 40 mM ammonium sulfate or 7 mM alanine as nitrogen source and 2% glucose. Cells were treated with formaldehyde (1% final concentration) for 20 min at 37°C and then glycine (125 mmol/L final concentration) for 5 min at 37°C. Formaldehyde-treated cells were harvested by centrifugation, washed with Tris-buffered saline, and then incubated in Buffer Z2 (1 mol/L Sorbitol, 50 mmol/L Tris-Cl at pH 7.4, 10 mmol/L β-mercaptoethanol) containing 2.5 mg of zymolase 20T for 20 min at 30°C on rocker platform. Spheroplast were pelleted by centrifugation at 3000*g*, and resuspended in 1.5 ml of NPS buffer (0.5 mmol/L Spermidine, 0.075% NP-40, 50 mmol/L NaCl, 10 mmol/L Tris pH 7.4, 5 mmol/L MgCl_2_, 1 mmol/L CaCl_2_, 1 mmol/L β-mercaptoethanol). Samples were divided into three 500 μl aliquots that were then digested with 22.5 U of MNase (Nuclease S7 from Roche) at 50 min at 37°C. Digestions were stopped with 12 μl of Stop buffer (50 mmol/L EDTA and 1% SDS) and were treated with 100 μg of proteinase K at 65°C over night. DNA was extracted twice by phenol/chloroform and precipitated with 20 μl of 5 mol/L NaCl and equal volume of isopropanol for 30 min at -20°C. Precipitates were resuspended in 40 μl of TE and incubated with 20 μg RNase A for 1 h at 37°C. DNA digestions were separated by gel electrophoresis from a 1.5% agarose gel. Monosomal bands (150 bp) were cut and purified by Wizard SV Gel Clean-Up System Kit (Promega, REF A9282). DNA samples were diluted 1:30 and used in quantitative polymerase chain reactions (qPCR) to quantify the relative MNase protection of each *LkALT1* and *KlALT1* template. qPCR analysis was performed using a Corbett Life Science Rotor Gene 6000 machine. The detection dye used was SYBR Green (2× KAPA SYBR FAST qBioline and Platinum SYBR Green from Invitrogen). Real-time PCR was carried out as follows: 94° for 5 min (1 cycle), 94° for 15 s, 58° for 20 s, and 72° for 20 s (35 cycles). Relative protection was calculated as a ratio to the control *LkVCX1*, and *KlVCX1* template found within a well-positioned nucleosome in +250 bp of the ORFs. The PCR primers amplify from around -950 to +250 bp (Supplementary Tables S2, S3) of *LkALT1* and *KlALT1* locus whose coordinates are given relative to the ATG (+1).

### Statistical Analysis

All the experiments were repeated two or three times. Data were analyzed statistically using unpaired *t*-test with Welch’s correction using Prism version 7.0c computer software. P values of 0.1 or less were considered statistically significant.

## Results

### *L. kluyveri LkALT1* and *K. lactis KlALT1* Encoded Enzymes Contribute to Alanine Metabolism

**FIGURE 2:**
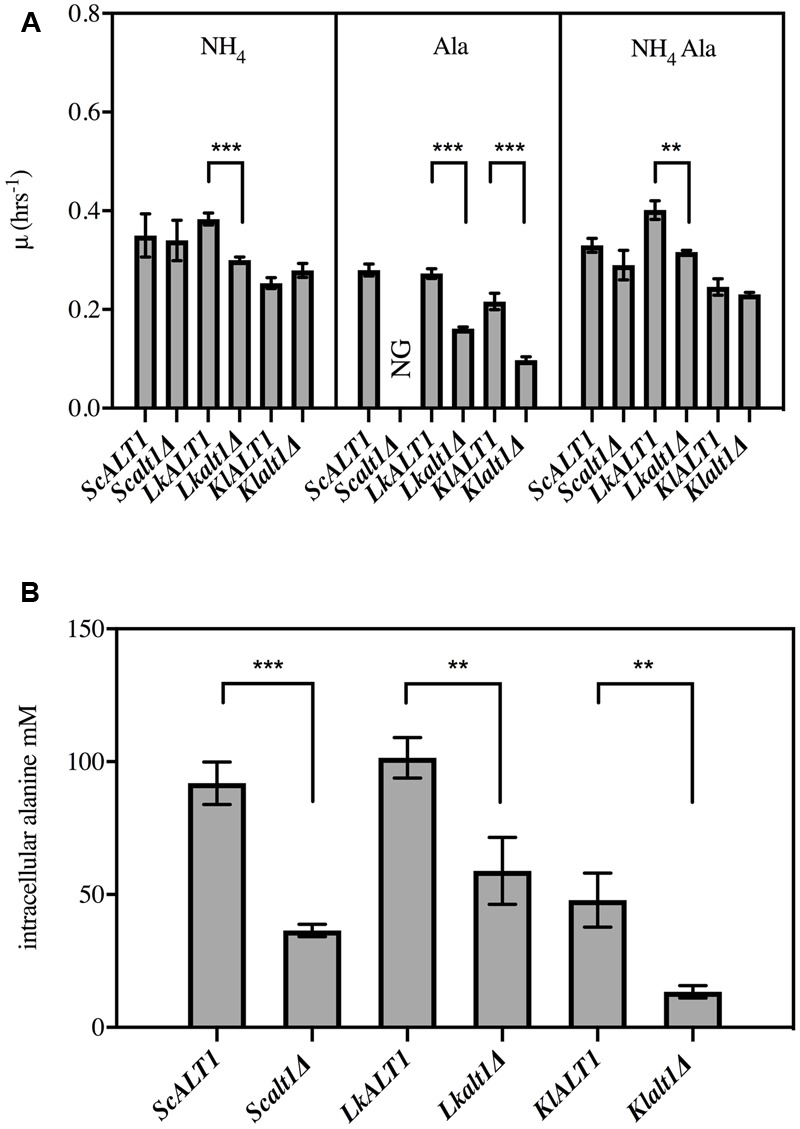
*Saccharomyces cerevisiae ScALT1, Lacchancea kluyveri LkALT1*, and *Kluyveromyces lactis KlALT1* are involved in alanine metabolism. **(A)** growth rate analysis of *S. cerevisiae, L. kluyveri*, and *K. lactis* wild type strain and single mutants was performed using glucose as carbon source and as sole nitrogen source ammonium sulfate, alanine, or ammonium sulfate plus alanine. Specific growth rate was determined during exponential phase. Values are presented as means ± SD from three independent experiments. NG, no growth. The asterisks indicates significantly different (^∗^*p* < 0.1; ^∗∗^*p* < 0.05; ^∗^*p* < 0.01) compared with the wt control. **(B)** Intracellular concentration of alanine in extracts obtained from glucose ammonium grown cells. After cells were grown and harvested during exponential phase, cell free extracts were prepared and alanine pools were determined, values are presented as means ± SD from two or three independent experiments. NG, no growth. The asterisks indicate significantly different (^∗^*p* < 0.1; ^∗∗^*p* < 0.05; ^∗^*p* < 0.01) compared with the wt control.

To address whether *L. kluyveri LkALT1* and *K. lactis KlALT1* preformed similar physiological roles to that of *Sc*Alt1, an analysis of its contribution to alanine metabolism was assessed by phenotypic analysis, through the obtention and phenotypic analysis of pertinent null mutants (**Figure 2A**). In a biosynthetic medium, with ammonium as nitrogen source, *Lkalt1Δ* displayed a 20% lower growth rate than the one observed for the wild type strain, surprisingly, alanine addition to the medium did not restore wild type growth rate. In a catabolic condition, when alanine was provided as sole nitrogen source, *Lkalt1Δ* growth rate was near half to that observed for the wild type strain; such growth reduction was not alleviated when ammonium sulfate was added to the growth medium, indicating that the role played by *Lk*Alt1 is not fully restored neither with alanine nor with ammonium sulfate, this observation will be discussed further. Conversely, in a biosynthetic medium *K. lactis Klalt1Δ* null mutant displayed wild type phenotype, and the braditrophic phenotype observed on alanine as sole nitrogen source was fully restored in the presence of both ammonium sulfate and alanine. These results indicate that, as well as for *ScALT1*, the orthologous genes *LkALT1* and *KlALT1* are involved in alanine catabolism. Nevertheless, unlike in *S. cerevisiae*, in *L. kluyveri* and *K. lactis* there is an alternative pathway to catabolize alanine. The growth rate displayed by *Lkalt1Δ* and *Klalt1Δ* in a biosynthetic medium suggests that as well as for *S. cerevisiae*, there is an independent pathway(s) for alanine biosynthesis, hampering the possibility to observe *LkALT1* and *KlALT1* contribution to alanine biosynthesis by growth rate analysis.

To further analyze the contribution of *LkALT1* and *KlALT1* to alanine biosynthesis, intracellular concentrations of this amino acid were determined in wild type and single mutants *Scalt1Δ, Lkalt1Δ* and *Klalt1Δ* during early exponential growth phase (OD_600__nm_ 0.3–0.6) on biosynthetic medium. To estimate alanine intracellular concentrations, a cell volume of 42 fl was considered for the three strains under study (Jorgensen et al., 2002; Klis et al., 2014). As reported earlier, *Sc*Alt1 contributed with 75–60% of the alanine pool, whereas the *Sc*Alt1-independent pathway only afforded 25–40% (Peñalosa-Ruiz et al., 2012). In *Lkalt1Δ* and *Klalt1Δ* strains a decrease of 42 and 70% in the intracellular alanine concentration, as compared to that found in the pertinent wild type strains, was observed (**Figure 2B**). In summary, presented results established that *LkALT1* and *KlALT1* are involved in alanine biosynthesis and catabolism, and that as well as for *S. cerevisiae*, there are alternative pathway(s) involved in alanine biosynthesis, which respectively provide 58 and 30% of the alanine pool in *L. kluyveri* and *K. lactis*, respectively. In regard to alanine catabolism, *Lkalt1Δ* and *Klalt1Δ* achieve 50% growth rate as compared with the one achieved in the wild type strain, indicating that as opposed to what has been observed in *S. cerevisiae*, in *L. kluyveri* and *K. lactis*, there are additional alanine catabolic pathways.

### *LkALT1* and *KlAL1* Are the Functional Orthologs of *ScALT1*

**FIGURE 3:**
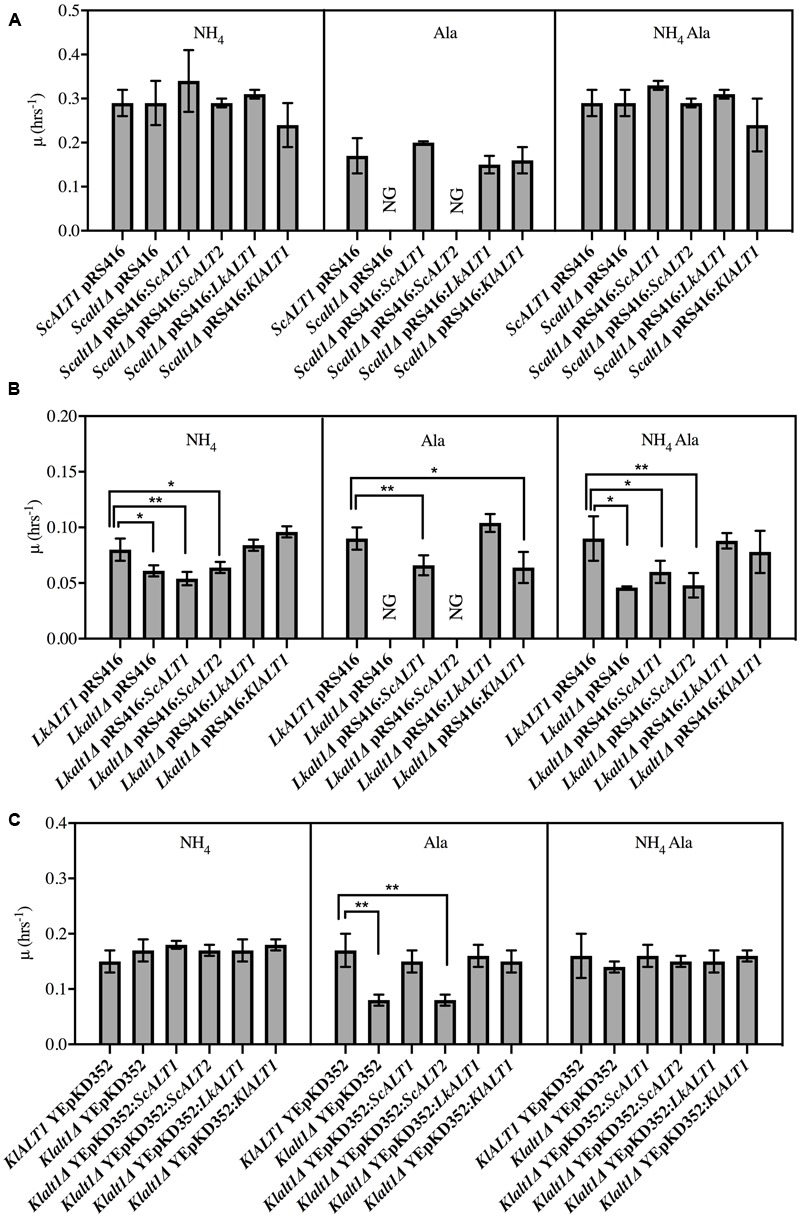
ScALT1, *LkALT1*, and *KlALT1* can substitute each other under catabolic conditions. Reciprocal complementation test was performed using glucose as carbon source and as sole nitrogen source ammonium sulfate, alanine or ammonium sulfate plus alanine. Specific growth rate was determined during exponential phase for **(A)** *S. cerevisiae*, **(B)** *L. kluyveri*, and **(C)** *K. lactis* pertinent transformed cells. For ectopic expression, the plasmid pRS416 was used for *S. cerevisiae* and *L. kluyveri* and the YEpKD352 for *K. lactis.* Values are presented as means ± SD from three independent experiments. NG, no growth. The asterisks indicate significantly different (^∗^*p* < 0.1; ^∗∗^*p* < 0.05; ^∗^*p* < 0.01) compared with the wt control. NG, no growth.

Above presented results indicate that *Sc*Alt1, *Lk*Alt1, and *Kl*Alt1 have close physiological roles. To analyze whether the three enzymes have an orthologous function, growth rate analysis of clones obtained from reciprocal complementations was performed (**Figure 3**). In a catabolic condition, where alanine was present as sole nitrogen source, transformation of an *Scalt1Δ* mutant with plasmids carrying either *ScALT1, LkALT1*, or *KlALT1* restored wild type growth (**Figure 3A**). Under the same culture conditions, *Lkalt1Δ* mutant strain was only partially complemented with plasmids carrying either *ScALT1* or *KlALT1*, while it was fully complemented when transformed with *LkALT1* (**Figure 3B**). When a *Klalt1Δ* mutant strain was transformed with plasmids harboring either *ScALT1, LkALT1*, or *KlALT1* wild type growth was attained (**Figure 3C**). As expected, *ScALT2* was unable to complement neither one of the three mutants tested (**Figures 3A–C**). This result indicates that *Lk*Alt1 and *Kl*Alt1 catabolic character has been maintained by *ScALT1*, conversely *ScALT2* has completely lost the capacity to fulfill *ScALT1, LkALT1*, or *KlALT1*catabolic role.

**FIGURE 4:**
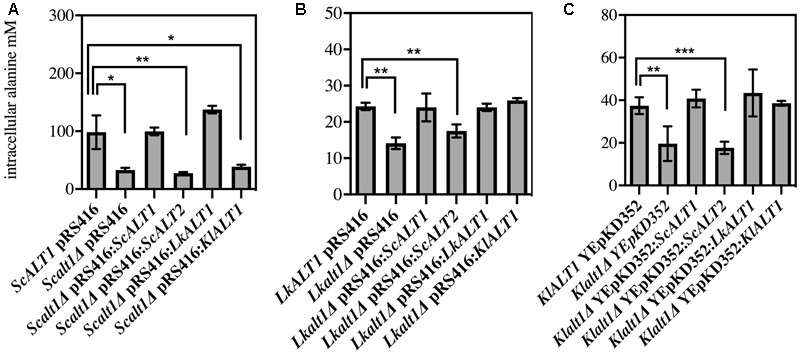
Reciprocal complementation test under biosinthetic conditions. Intracellular concentration of alanine from **(A)** *S. cerevisiae*, **(B)** *L. kluyveri* and **(C)** *K. lactis* transformed strains was obtained from glucose ammonium grown cell extracts. After cells were grown and harvested during exponential phase, cell free extracts were prepared and alanine pools were determined. Values are presented as means ± SD from two-three independent experiments. The asterisks indicates significantly different (^∗^*p* < 0.1; ^∗∗^*p* < 0.05; ^∗^*p* < 0.01) compared with the wt control.

As shown in **Figure 2** the only mutant with a decreased growth rate in a biosynthetic condition was *Lkalt1Δ.* When it was transformed with plasmids harboring either *LkALT1* or *KlALT1* wild type growth rate was attained, but neither *ScALT1* nor *ScALT2* were able to restore the growth rate observed in the wild type transformed with an empty plasmid (**Figure 3B**). *Scalt1Δ* and *Klalt1Δ* mutant strains, did not show a growth defect in the presence of ammonium sulfate as sole nitrogen source. In order to analyze their biosynthetic role, alanine pools were determined in extracts prepared from the mutant strains transformed with plasmids bearing the pertinent genes, as controls wild type and mutants transformed with empty plasmids were used (**Figure 4**), all extracts were prepared from exponential growth phase (OD_600__nm_ 0.3–0.6). Both *Sc*Alt1 and *Lk*Alt1 were able to restore alanine intracellular wild type concentrations in *Scalt1Δ, Lkalt1Δ* and *Klalt1Δ* mutant strains (**Figures 4A–C**). *Lkalt1Δ* and *Klalt1Δ* transformed with *KlALT1* displayed a wild type alanine intracellular concentration (**Figures 4B,C**). Nevertheless, *Kl*Alt1 failed to reestablish the wild type alanine concentration of the *Scalt1Δ* mutant (**Figure 4A**). The inability of *Kl*Alt1 to restore *Scalt1Δ* alanine pool when grown under biosynthetic conditions could be due to peculiar kinetic properties of *KlALT1*-encoded protein and/or to a low transcriptional rate of *KlALT1.* As expected, all mutant strains transformed with plasmids harboring *ScALT2* presented an equivalent alanine intracellular concentration to that found in the negative controls.

These results, show that *Sc*Alt1, *Lk*Alt1, and *Kl*Alt1 can fully or partially substitute each other when alanine or ammonium sulfate is present as sole nitrogen source, confirming that *Sc*Alt1 has retained enough ancestral characteristics to conclude that *Lk*Alt1 and *Kl*Alt1 are the functional orthologs of *Sc*Alt1 with respect to its performance in both alanine biosynthesis or catabolism, conversely, *Sc*Alt2 has lost its role in alanine metabolism.

It is important to highlight the observation that in a medium provided with ammonium sulfate as sole nitrogen source *Lkalt1Δ* with or without an empty plasmid displayed a growth defect (**Figures 2, 3B**) and wild type growth phenotype was not attained when neither alanine was added to the medium (**Figure 2**) nor when *Lkalt1Δ* was transformed with a plasmid harboring *ScALT1* (**Figure 3B**). Although, *Lkalt1Δ* transformed with *ScALT1*, displayed wild type alanine intracellular concentration (**Figure 4B**), indicating that *Lkalt1Δ* growth defect in a biosynthetic condition was not due to a lower alanine intracellular concentration. Taken together these observations provide strong experimental evidence to sustain that *LkALT1* has a yet unidentified alternative function that is also present in *KlALT1*, since plasmids carrying *KlALT1* not only restored *Lkalt1Δ* alanine pool (**Figure 4B**), but also complemented the growth phenotype under the biosynthetic condition tested (**Figure 3B**). Worth of mention is the fact that in *K. lactis, KlALT1* alternative function does not result in any apparent phenotype when *Klalt1Δ* mutant is grown on either ammonium sulfate or alanine as nitrogen sources. The observation that *KlALT1* complements *Lkalt1Δ* decreased growth on ammonium sulfate and alanine uncovers the fact that *Kl*Alt1 harbors an equivalent function to that observed in *Lk*Alt1. Furthermore, both *ScALT1* and *ScALT2* were unable to reestablish wild type growth rate of *Lkalt1Δ* strain in a medium with ammonium sulfate and alanine as nitrogen sources (**Figure 3B**), indicating that neither *ScALT1* nor *ScALT2* can substitute the supplementary function present in *LkALT1* (**Figures 2A, 3B**).

### *Sc*Alt1, *Lk*Alt1, and *Kl*Alt1 Exhibit Alanine Transaminase Activities

To compare the *Sc*Alt1, *Lk*Alt1, and *Kl*Alt1 transaminase activities displayed during biosynthetic and catabolic conditions, extracts obtained during exponential phase (OD_600__nm_ 0.4–0.6) from *S. cerevisiae, L. kluyveri*, and *K. lactis* wild type strains, grown in glucose-ammonium sulfate and glucose-alanine, were tested for alanine transaminase activity. Extracts obtained from *Scalt1Δ, Lkalt1Δ*, and *Klalt1Δ* mutant strains were used as negative controls (**Table 2**). In agreement with a previous report from our group (Peñalosa-Ruiz et al., 2012), *Sc*Alt1 and *Lk*Alt1 alanine transaminase specific activity increased approximately 10-fold in cultures provided with alanine as sole nitrogen source as compared to those grown on ammonium sulfate, suggesting alanine could play and activator role. In *K. lactis* alanine transaminase activity was equivalent in samples prepared from cultures grown on either alanine or ammonium sulfate as nitrogen sources. Unexpectedly, alanine transaminase activity was detected in extracts of the mutant strains *Lkalt1Δ* and *Klalt1Δ* grown in either ammonium or alanine as sole nitrogen sources (**Table 2**). When the extracts came from ammonium sulfate the activity displayed by the mutant *Lkalt1Δ* was similar to that observed in the *L. kluyveri* wild type strain. However, in extracts prepared from cells grown on alanine as sole nitrogen source, mutant strain *Lkalt1Δ* activity was 20 times lower than that observed in the wild type strain (0.12 vs. 2.77 μmoles min^-1^ mg protein^-1^), although it was only 2.5-fold lower to that one observed in extracts from ammonium sulfate (0.12 vs. 0.28 μmoles min^-1^ mg protein^-1^), suggesting the existence of an additional alanine transaminase. Similarly, the specific activity detected in extracts from the mutant *Klalt1Δ* coming from ammonium or alanine was smaller than the observed in the wild type (0.024 vs. 0.036 μmoles min^-1^ mg protein^-1^), again suggesting the existence of a second alanine transaminase in *L. kluyveri* and *K. lactis*. It can be thus concluded that *ScALT1, LkALT1*, and *KlALT1* encoded proteins display alanine transaminase activity in the three yeasts under study and that *L. kluyveri* and *K. lactis* alanine transaminase activity is not exclusively encoded by *LkALT1* and *KlALT1*.

**Table 2:** Alanine transaminase specific activity in extracts prepared from glucose–ammonium sulfate or glucose-alanine grown cultures.

	Glucose	
	NH_4_	L-alanine
*ScALT1*	0.093 ± 0.001	0.821 ± 0.093
*Scalt1Δ*	0.018 ± 0.001	NG
*LkALT1*	0.259 ± 0.014	2.771 ± 0.399
*Lkalt1Δ*	0.289 ± 0.142	0.120 ± 0.031
*KlALT1*	0.036 ± 0.003	0.036 ± 003
*Klalt1Δ*	0.024 ± 0.003	0.024 ± 0.002

*Specific activity is expressed as μmol min^-*1*^ mg of protein^-*1*^. No growth (NG). Values are presented as mean from three mesurments ± SD. NG, no growth.*

### Determination of Alanine Transaminase Activity in Complemented Strains

**FIGURE 5:**
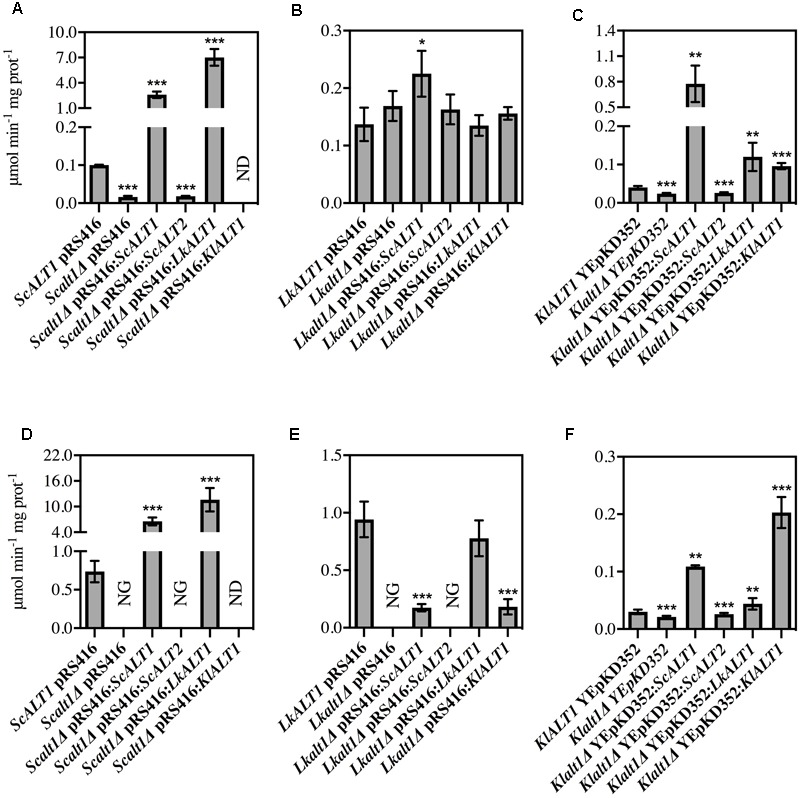
Alanine transaminase specific activity in extracts prepared from complemented strains. Cellular extracts from **(A)** *S. cerevisiae*, **(B)** *L. kluyveri*, and **(C)** *K. lactis* in glucose- ammonium sulfate or **(D–F)** in glucose–alanine grown cultures. Specific activity is expressed as μmol min^-1^ mg of protein^-1^, values are presented as means ± SD from three independent experiments. NG, no growth; ND, not detected. The asterisks indicate significantly different (^∗^*p* < 0.1; ^∗∗^*p* < 0.05; ^∗^*p* < 0.01) compared with the wt control.

To confirm the orthologous function of the three studied enzymes, alanine transaminase activity was also tested in *Scalt1Δ, Lkalt1Δ* and *Klalt1Δ* mutants transformed with plasmids carrying either *ScALT1, ScALT2, LkALT1*, or *KlALT1* in either glucose ammonium sulfate or glucose alanine, as controls wild type and mutants transformed with empty plasmids were used (**Figure 5**). In many cases, it was observed that extracts from the mutant strains transformed with plasmids harboring either *ScALT1, LkALT1* or *KlALT1* exhibited higher specific activity, than the one observed in the wild type strains in each one of the conditions tested (**Figures 5A,C,D,F**). These results could be attributed to both a difference in the transcriptional regulation of the heterologous enzymes or to the plasmid copy number, since YEpKD352 is a high copy number plasmid and pRS416 is a centromeric (*CEN*) plasmid, of which one or two copies are maintained per cell (Sikorski and Hieter, 1989), thus, increased enzymatic activity can be expected. As expected the *Scalt1Δ* mutants transformed with plasmids carrying either *ScALT1* or *LkALT1* displayed higher specific activity than the wild type strain in extracts from either glucose-ammonium or glucose-alanine (**Figures 5A,D**). As in the wild type strain, the specific activity of these transformed strains was higher in extracts coming from alanine supplemented medium than from ammonium sulfate. The specific activity of extracts from *Scalt1Δ* transformed with *KlALT1* were not detectable in neither one of the conditions tested (**Figures 5A,D**). However, as described earlier, plasmids harboring *KlALT1* were able to functionally substitute *ScALT1* in medium with alanine as sole nitrogen source (**Figure 3A**), suggesting that lack of alanine transaminase activity in the extracts prepared from this strain grown on either glucose-ammonium sulfate or glucose-alanine was due to a an in vitro artifact which prevents detection of activity in these extracts. When grown in a medium with ammonium sulfate as sole nitrogen source, extracts from *Lkalt1Δ* transformed with an empty plasmid, presented equivalent alanine transaminase specific activity to that found in extracts of its corresponding wild type strain (**Figure 5B**). As observed before, in *L. kluyveri* alanine transaminase activity was not exclusively displayed by *Lk*Alt1 (**Table 2**). Thus, the activity measured under this condition, in all the *Lkalt1Δ* transformed strains, is the result of the combined activities of the heterologous enzyme and the alternative alanine transaminase. Even though this other transaminase activity is also present in extracts of *Lkalt1Δ* grown in alanine, its contribution is minor, not allowing growth of *Lkalt1Δ* carrying pRS416 empty plasmid. In this medium extracts from *Lkalt1Δ* transformed with *LkALT1* presented an activity similar to that detected in extracts obtained from the wild type strain, while those obtained from *Lkalt1Δ* transformed with *ScALT1* or *KlALT1*, presented a four times lower activity than the one observed in the wild type strain (**Figure 5E**), consistent with the partial complementation phenotype of these strains (**Figure 3B**). The activity in extracts obtained from glucose–ammonium sulfate or glucose–alanine of *Klalt1Δ* transformed with *ScALT1, LkALT1*, or *KlALT1* was higher than in the wild type. All the mutants transformed with plasmids harboring *ScALT2* did not display activity as well as the negative controls (**Figures 5A–F**), indicating *ScALT2* incapacity to complement *ScALT1, LkALT1*, and *KlALT1* (**Figures 3, 4**). This result was expected since we had previously observed that even when *ScALT2* is over-expressed using a *TET* promoter, higher production of *Sc*Alt2 protein does not lead to detectable activity (Peñalosa-Ruiz et al., 2012). Increased activity of some samples, due to plasmid copy number, as compared to their respective wild type strain was not followed by an increased alanine pool or higher growth rate (**Figures 3, 4**), indicating that an increase in the enzyme concentration, does not result in higher alanine production, suggesting that *in vivo* these enzymes, work far from their Vmax, so increased enzyme concentration does not result in a higher flux through this enzymes.

### *Lk*Alt1 and *Kl*Alt1 Display Similar Kinetic Parameters and a Ping Pong Mechanism

To investigate *Lk*Alt1 and *Kl*Alt1 kinetic properties, His-tagged enzymes were purified to electrophoretic homogeneity after heterologous over-expression in *E. coli*. Denaturing SDS-PAGE showed a molecular mass of about 50 kDa for the two enzymes, in agreement with the value deduced from their amino acid sequences, which were 61.16 and 59.23 kDa for *Lk*Alt1 and *Kl*Alt1, respectively (Supplementary Figure S1). The oligomeric structures revealed by light scattering of the purified samples (*Lk*Alt1 100.1 ± 10.8 kDa and *Kl*Alt1 135.9 ± 25.8 kDa) indicate that the active form of both enzymes is an homodimer. Enzymatic activity of the pure enzymes was measured in Tris-HCl buffer at pH values ranging from 5.5 to 8.5, maximum activity was obtained around pH 7 for the two enzymes. It is worth mentioning that *Lk*Alt1 was sensitive to acidic pH while *Kl*Alt1 was sensitive to basic pH (Supplementary Figure S2).

**FIGURE 6:**
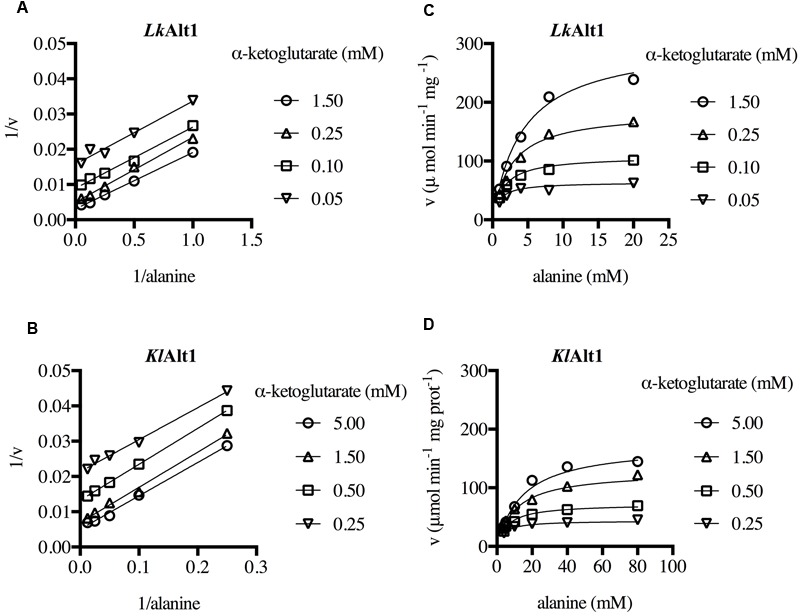
*Lk*Alt1 and *Kl*Alt1 are alanine transaminases with the characteristic ping pong mechanism. Initial velocities were measured at different alanine and α-ketoglutarate concentrations. Double reciprocal plots of **(A)** *Lk*Alt1 and **(B)** *Kl*Alt1. Global fit of **(C)** *Lk*Alt1 and **(D)** *Kl*Alt1 to the ping pong equation. The corresponding kinetic parameters are shown in **Table 3**.

Initial velocity measurements were made at different α-ketoglutarate and alanine concentrations. The resulting double reciprocal plot pattern obtained for both enzymes, corresponded to that expected for enzymes with a Ping-Pong mechanism (**Figures 6A,B**). Thus, the data were globally fitted to a Ping-Pong equation (**Figures 6C,D**). The resulting kinetic parameters for *Lk*Alt1 and *Kl*Alt1 are presented in **Table 3**. It follows that the orthologous proteins *Lk*Alt1 and *Kl*Alt1 are alanine transaminases with similar *k*_cat_, and *k*_m_, in both cases the affinity for α-ketoglutarate was higher than the one for alanine (**Table 3**). Results from our laboratory indicate that *S. cerevisiae Sc*Alt1 is also an alanine transaminase (Peñalosa-Ruiz et al., 2012) with close kinetic parameters to the ones observed in *L. kluyveri Lk*Alt1 and *K. lactis Kl*Alt1 (unpublished results).

**Table 3:** Kinetic parameters of *L. kluyveri Lk*Alt1 and *K. lactis Kl*Alt1.

Enzyme	*k*_cat_ (s^-1^)	*k*_a_ (mM)	*k*_b_ (mM)	*R*^2^
*Lk*Alt1	355	4.88	0.22	0.98
*Kl*Alt1	203	17.25	0.92	0.98

*Data were fitted to the ping pong equation. *k*_*a*_ represents the *k*_*m*_ for alanine and *k*_*b*_ the *k*_*m*_ for α-ketoglutarate.*

### Divergent Expression Profile between *LkALT1* and *KlALT1* Is Independent of Chromatin Organization

**FIGURE 7:**
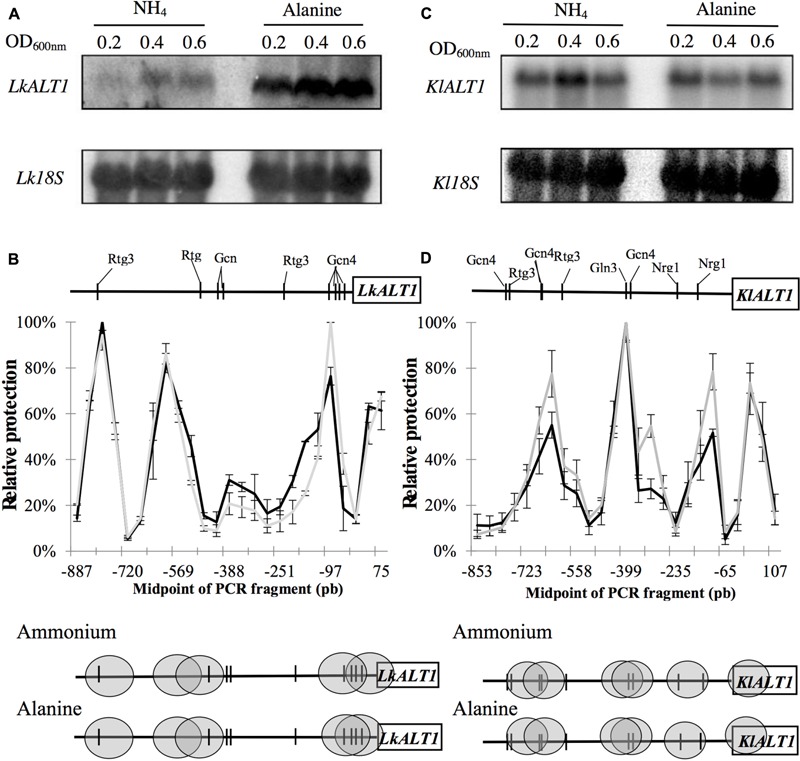
*LkALT1* and *KlALT1* expression profile and NuSA assay. Northern blot analysis shown expression profile of *LkALT1* **(A)** and *KlALT1* **(B)**, in both cases *Lk18S* or *Kl18S* ribosomal RNA was used as control in glucose–amoniumm sulfate or glucose alanine. **(C)** Nucleosome scanning assay (NuSA) *LkALT1* gene promoter in glucose-ammonium (black line) or glucose–alanine (gray line); and **(D)** Nucleosome Scanning Assay (NuSA) *KlALT1* gene promoter in glucose–ammonium (black line) or glucose-alanine (gray line); nucleosomes are shown in gray ovals and vertical lines shown DNA binding sites. Error bars represent the standard deviations from two independent experiments.

In *S. cerevisiae, ScALT1* and *ScALT2* are differentially expressed. Previous analysis revealed that the expression profile of *ScALT1* resembles that of a catabolic gene, whose expression is induced in the presence of alanine. Opposed expression pattern is observed for *ScALT2*, which is repressed whenever alanine is present (Peñalosa-Ruiz et al., 2012). To investigate whether the expression pattern of *LkALT1* and *KlALT1* was similar to that of *ScALT1* or *ScALT2*, Northern blot analysis were carried out in samples obtained from wild type strain cultures in different growth phases on either glucose-ammonium sulfate or glucose-alanine. *LkALT1* was repressed in a medium with ammonium sulfate as sole nitrogen source and induced when alanine was the nitrogen source (**Figure 7A**), mirroring *ScALT1* expression profile (Peñalosa-Ruiz et al., 2012). *KlALT1* showed constitutive expression, was equally expressed in both glucose-ammonium sulfate and glucose-alanine conditions (**Figure 7C**). Enzymatic activity followed the expression profile observed for the two orthologous genes *LkALT1* and *KlALT1* (**Table 2**).

To analyze *LkALT1* and *KlALT1* promoter organization, nucleosome scanning assay (NuSA) was preformed. Samples obtained from exponential phase growth cultures (OD_600__nm_ 0.3) on ammonium sulfate or alanine as sole nitrogen sources were analyzed to determine nucleosome positioning across the promoters of both genes. After yeast DNA-nucleosome interaction was fixated with formaldehyde, cells were spheroplasted and incubated with MNase. The resulting DNA fragments obtained after MNase digestion were analyzed by quantitative PCR (qPCR). For normalization *L. kluyveri LkVCX1* locus (SAKL0F09878g), or *K. lactis KlVCX1* locus (KLLA-ORF439) (Infante et al., 2011) were used. Nucleosome positioning was equivalent for *LkALT1* and *KlALT1* promoters when cells were grown on ammonium sulfate or alanine (**Figures 7B,D**), indicating that the different expression profiles observed for the *LkALT1* promoter on ammonium sulfate or alanine was not related to nucleosome organization (**Figure 7A**). As expected for *KlALT1*, which displays constitutive expression, NuSA analysis showed similar nucleosome organization on both growth conditions (**Figure 7D**). Since the nucleosome positioning did not change in either one of the promoters tested it can be hypothesized that the most likely transcriptional factors involved in *LkALT1* and *KlALT1* transcriptional regulation should have their binding sites in the Nucleosome Free Region (NFR). Accordingly, an “*in silico*” analysis of the *LkALT1* and *KlALT1* promoter sequences was performed^1^, considering the consensus and conserved binding elements known for *S. cerevisiae*. In both promoters, *cis* elements for Gcn4 and Rtg3 in either free or nucleosome protected regions were found, additionally, in *KlALT1* promoter region an Nrg1 sequence was found. This analysis suggests that *LkALT1* could be regulated by the combined action of Gcn4 and Rtg3 orthologous transcriptional factors, while that of *KlALT1* would also combine Nrg1. Further research on this matter will be required to determine whether the predicted regulators are involved in *LkALT1* and *KlALT1* transcriptional regulation.

## Discussion

Here, we present a combined phenotypic, biochemical and transcriptional regulation analysis of the *L. kluyveri LkALT1* and *K. lactis KlALT1* genes, which shows that both encode transaminases involved in alanine biosynthesis and catabolism. Interestingly, the *S. cerevisiae* isozyme encoded by *ScALT1*, retained alanine transaminase activity, while *ScALT2* encoded enzyme did not conserve this activity (Peñalosa-Ruiz et al., 2012). In all three cases transaminase activity followed gene expression levels of their encoding genes. Accordingly, while *ScALT1, LkALT1* and *KlALT1*, were capable of reciprocally complementing their role in alanine metabolism, *ScALT2* was not. Moreover, it was discovered that *LkALT1* and *KlALT1* harbors an additional function besides that of alanine transaminase, which was neither retained by *ScALT1* nor *ScALT2*.

### Insights into the Evolution of Alanine Metabolism

Of the three yeasts, which were used as experimental models in this work, *K. lactis* was the first one to diverge from the linage leading to *S. cerevisiae* (Merico et al., 2007; Huerta-Cepas et al., 2014; Marcet-Houben and Gabaldón, 2015), thus it can be considered that this yeast is an appropriate representative of *KLE* progenitor physiology. In *K. lactis* alanine metabolism is accomplished by more than one pathway; trough the *Kl*Alt1 alanine transaminase, and must likely by another yet unidentified alanine transaminase. Nevertheless, the existence of alternative pathways involved in alanine metabolism cannot be ruled out for example glutamine transaminase, which is known to exist in *S. cerevisiae* (Soberón and González, 1987), that could support alanine biosynthesis from pyruvate and glutamine. In *K. lactis*, the expression of *KlALT1* and the activity displayed by the encoded protein is equivalent under biosynthetic or catabolic conditions. Also, the *Kl*Alt1 alanine transaminase activity detected in a *Klalt1Δ* mutant shows similar enzymatic activity in both conditions, suggesting that *Kl*Alt1 is similarly specialized in alanine biosynthesis and catabolism. Nevertheless, while a *Klalt1Δ* strain displays wild type growth rate under biosynthetic conditions, it shows decreased growth rate when grown on alanine as sole nitrogen source, indicating that the alternative pathways involved in alanine metabolism cannot fully compensate *Kl*Alt1catabolic role.

As well as *K. lactis, L. kluyveri* can also be considered a *KLE* ancestral type yeast. In *L. kluyveri*, alanine biosynthesis and catabolism can be achieved by more than one pathway, since an *Lkalt1Δ* mutant is able to grow on ammonium or alanine as sole nitrogen sources. In *L. kluyveri LkALT1* expression is increased when alanine is provided as sole nitrogen source, which results in higher alanine transaminase activity, constituting a specialization of *Lk*Alt1 catabolic role. In addition, under this condition, the *Lk*Alt1 independent alanine transaminase decreases its activity, enhancing the leading role of *Lk*Alt1 in alanine catabolism. The expression pattern observed in *LkALT1*, favoring its catabolic role, was maintained by *ScALTI*, but the degree of specialization in alanine catabolism increased to the extent in which *Sc*Alt1 is the only enzyme capable of catabolizing alanine, since the alternative pathway for alanine catabolism present in *K. lactis* and *L. kluyveri* was lost in *S. cerevisiae*. It is not surprising that the specialization of *Sc*Alt1 and *Lk*Alt1 towards alanine catabolism was achieved through a change in gene expression pattern, since the reaction catalyzed by transaminases, is close to equilibrium, and thus the direction of the reaction largely depends on substrates and products concentration, and given the enzymatic mechanism of the reaction, modification in the kinetic parameters of the enzyme would affect in the same proportion the biosynthetic and catabolic direction of the reaction (Nisonoff et al., 1953). It could be considered that the catabolic specialization present in these enzymes is compelling in order to improve their anaplerotic character which enables them to furnish pyruvate to the tricarboxilic acid cycle (Land, 2002). It can be considered that the anaplerotic role of alanine transaminase is less compelling for *K. lactis* than for *S. cerevisiae* and *L. kluyveri*, since *K. lactis* metabolism is constitutively respiratory (Breunig et al., 2000) and *S. cerevisiae* and *L. kluyveri* have a facultative metabolism (Moller et al., 2002; Merico et al., 2007). Hence, *L. kluyveri* and *S. cerevisiae* catabolic specialization arrived to the extreme in which *S. cerevisiae* lost the alternative pathways for alanine catabolism present in both *L. kluyveri* and *K. lactis*. However, increased catabolic character did not affect the prominent biosynthetic role played by these three enzymes, which contribute with at least half of the alanine pool, even when more than one pathway for alanine biosynthesis is present in the three species. This redundancy is also found in *E. coli*, where alanine is synthesized by three different transaminases, and only the triple mutant is an alanine auxotroph (Yoneyama et al., 2011). In this regard, it is important to consider that in *E. coli* alanine is the second amino acid with highest intracellular concentration, and in *Neurospora* and *S. cerevisiae* it is the amino acid displaying highest intracellular concentration (Yuan et al., 2006; Canelas et al., 2008; Kim et al., 2011). Although no information on free intracellular amino acid pools exists for *L. kluyveri* and *K. lactis*, results presented in this paper indicate that alanine could constitute one of the highest accumulated amino acids in these yeasts. These observations support the proposition that alanine could play an important role in addition to that as protein building block as has been shown for other amino acids (Stephen and Jamieson, 1997; Takagi et al., 1997; Crespo and Hall, 2002; Eisler et al., 2004; Binda et al., 2009).

### *ScALT2* Evolutionary History

Although the closest taxonomic group of *ScALT2* parental strain is the *KLE* clade, *Sc*Alt2 is not an alanine transaminase, nor displays the alternative function encoded by *LkALT1* and *KlALT1.* A structural analysis comparing *Sc*Alt1 and *Sc*Alt2 sequences demonstrated that *Sc*Alt2 has conserved the binding site for alanine and for pyridoxal-5-phospate which is the coenzyme which enables transaminase activity (García-Campusano, 2009). Together, these data indicate that in the *KLE* parental strain the ancestral form of *ScALT2* codified for an alanine transaminase. Thus, it can be considered that when the hybrid was formed the *KLE* parental strain contributed with at least two alanine transaminases, one of them endosed by the ancestral form of *ScALT2*, and another one codified by a yet unidentified gene encoding the observed transaminase activity present in the *Lkalt1Δ* and *Klalt1Δ* mutants. In addition, the *ZT* parental strain provided a third alanine transaminase, which corresponded to the *ScALT1* ancestral form, which probably was already specialized towards alanine catabolism through gene expression, since this tendency is already present in *L. kluyveri LkALT1.* The specialization of *ScALT1* towards alanine catabolism and its contribution to alanine biosynthesis, not only relaxed the selective pressure that would favor the maintenance of an *ScALT1* and *ScALT2* independent alternative catabolic pathways, provided by the *KLE* parental, but also flexibilized the need to conserve *ScALT2* encoded alanine transaminase activity. Hence, the divergence between *ScALT1* and *ScALT2* can be explained by loss-of-function, sub-functionalization, and neofunctionalization models posed to explain the fate of duplicated genes. The scenario provided by the loss-of-function model, is the most unlikely, since *ScALT2* promoter has retained the capacity to respond to alanine concentration and a complete soluble protein with no transaminase activity is synthesized (Peñalosa-Ruiz et al., 2012). If the divergence of *ScALT1* and *ScALT2* had occurred through subfunctionalization, one would expect that the transaminase activity present in *Lk*Alt1 and in *Kl*Alt1 would have only been retained by *Sc*Alt1 and lost by *Sc*Alt2, as shown in this work; whereas, the alternative function provided by *LkALT1* and *KlALT1* would have been delegated to *ScALT2* and lost from *ScALT1*, which is not consistent with the finding that *Sc*Alt2 does not complement neither *Lkalt1Δ* nor *Klalt1Δ* mutants. Finally, under a neofunctionalization model, it would be expected that *Sc*Alt2 function would be absent from either *ScALT1 LkALT1* or *KlALT1.* The data herein presented support this later scenario*;* nevertheless, further studies are needed to understand the role of *Sc*Alt2 and to distinguish between the above presented scenarios.

## Author Contributions

AG designed experiments, wrote the MS, obtained funding. XE-F performed and designed experiments, wrote the MS. CC-B performed experiments. MC performed and designed experiments, contributed to MS writing. JG performed experiments. DM performed experiments.

## Conflict of Interest Statement

The authors declare that the research was conducted in the absence of any commercial or financial relationships that could be construed as a potential conflict of interest.

## Footnotes

1 www.yeastract.com
