# Supplementary material for: Evolutionary Diversification of Alanine Transaminases in Yeast: Catabolic Specialization and Biosynthetic Redundancy
Source: Front Microbiol. 2017 Jun 26;8:1150. doi: 10.3389/fmicb.2017.01150 (PMC5483587; doi:10.3389/fmicb.2017.01150)
Supplement: Supplementary file 1 [file Table_1.PDF]

Evolutionary Diversification of Alanine Metabolism in Yeast: Catabolic Specialization and Biosynthetic Redundancy

Ximena Martínez de la Escalera-Fanjul, Carlos Campero-Basaldua, Maritrini Colón, James González, Dariel Márquez, and Alicia González[1]*

*Author for correspondence:
Alicia González
amanjarr@ifc.unam.mx

Table S1. Oligonucleotides used in the present work

Name	Sequence	Application
X1	ATTTCATCTCACTTTCTTCTCGC AACACC	*Lkalt1*Δ 5´ deletion module
X2	CCC CTT CGT TCC CTA GTC ACT CCT TT**C GGG TTA ATT AAG GCG CGC CAG**	*Lkalt1*Δ 5´ deletion module, in bold 5’ end of the kanMX4 selection marker
X3	CGGGTT AAT TAA GGC GCG CCA G	*Lkalt1*Δ *kanMX4* deletion module
X4	CCAGTGTCGAAAACGAGCTCG	*Lkalt1*Δ *kanMX4* deletion module
X5	**CCAGTGTCGAAAACGAGCTC** GAGTCTCAGAACCTTTTTTTA AGACGCTTG	*Lkalt1*Δ 3´ deletion module, in bold 3’ end of the kanMX4 selection marker
X6	GCAGTCTAGGCAGGGATAGTAG GAATT	*Lkalt1*Δ 3´ deletion module
X7	ATTAGCAGAGTGAGTGTTGTCC CTC	*Lkalt1*Δ deletion module fusion
X8	CCATATTTCAAGCACATGCCCC	*Lkalt1*Δ deletion module fusion
X9	AAACCCTCCACCAGCATAAGTT C	*Lkalt1*Δ 5´ confirmation
X10	ATT ACG GCT CCT CGC TGC AG	*Lkalt1*Δ 5´ confirmation
X11	TGATTTTGATGACGAGCGTAAT	*Lkalt1*Δ 3´ confirmation
X12	GGGTGACAACCACACAAATCTA GAG	*Lkalt1*Δ 3´ confirmation

Name	Sequence	Application
X13	AAAGTACCATAACGTGAACCGG G	*Klalt1*Δ 5′ deletion module
X14	GCCTTAATTAACCCGGGGATCC GTCGACCTGCAGCGTACG**CAGT GTCAGAAGTGGGAGAATTCC**	*Klalt1*Δ 5′ deletion module, in bold 5' end of the kanMX4 selection marker
X15	**GGAATTCTCCCACTTCTGACA CTG**CGTACGCTGCAGGTCGAC	*Klalt1*Δ *kanMX4* deletion module, in bold 5' end of *KlALT1*.
X16	TTGAAGTGGATGATGAAGCATG CTCAGGCATACCCAGAAC**ATCG ATGAATTCGAGCTCGT**	*Klalt1*Δ *kanMX4* deletion module, in bold 3' end of *KlALT1*.
X17	**CGAGCTCGAATTCATCGAT**GT TCTGGGTATGCCTGAGCATGC	*Klalt1*Δ 3′ deletion module, in bold 3' end of the kanMX4 selection marker
X18	TATCAAGGGAACGACAACACGG ACG	*Klalt1*Δ 3′ deletion module
X19	GGCTTACTGCTGCTAAATCGC	*Klalt1*Δ 5′ confirmation
X20	ATTACGGCTCCTCGCTGCAG	*Klalt1*Δ 5′ confirmation
X21	GGCTCTGAGTAGATTCTCTTTGG	*Klalt1*Δ 3′ confirmation
X22	GCCTCGGTGAGTTTTCTCCT	*Klalt1*Δ 3′ confirmation
X23	**CGACGGTATCGATAAGCTTGA TATCG**GGCACCATTATCATCACC ATAC AAGTCG	pRS416:*ScALT1*, in bold pRS416 homologous region
X24	GCATCTTCGCTTCTCTATGCCAT G TTTCC**CACTAGTTCTAGAGCG GCC GCCA**	pRS416:*ScALT1*, in bold pRS416 homologous region
X25	GCGCGC**GGTACC**TGCTAGCGG CAC CATTATCATCACC	YEpKD352:*ScALT1*, in bold restriction site for KpnI
X26	CTTACCAATGGCACCTAACTTTG C **GTCGAC**GCGCGC	YEpKD352:*ScALT1*, in bold restriction site for SalI
X27	GCGCGC**GAATTC**GCTGAAGAC GTT CAACTCACC	pRS416:*ScALT2* and YEpKD352:*ScALT2*, in bold restriction site for EcoRI
X28	CATGCATCATTCTTTACTAAGGG **GGAT CC**GCGCGC	pRS416:*ScALT2* and YEpKD352:*ScALT2*, in bold restriction site for BamHI

Name	Sequence	Application
X29	GCAC**GAGCTC**CCAAGTATTTTT CATGTCAAGTC	pRS416:*LkALT1* and YEpKD:*LkALT1*, in bold restriction site for SacI
X30	CCATAAATTCAATTACAGTAAAG **GAGCTC**GTGC	pRS416:*LkALT1* and YEpKD:*LkALT1*, in bold restriction site for SacI
X31	CGCGCG **GGTACC**AATCGGCTAATT CGAGTACTGGC	pRS416:*KlALT1* and YEpKD:*KlALT1*, in bold restriction site for KpnI
X32	CGAATCATGATCAAGCAACACA TACG**G TCGAC**CGCGCG	pRS416:*KlALT1* and YEpKD:*KlALT1*, in bold restriction site for SalI
X33	GCGCGC**GCTAGC**ATGTATAAGT TCCAACCTGCTGATAAGC	*LkALT1* His-tag, in bold restriction site for NheI
X34	CCTTCTTTGACAAGTACCGTGAT TAG**CTCGAG**CGCGCG	*LkALT1* His-tag, in bold restriction site for XhoI
X35	GCGCGC**CATATG**ACTGTGAAAC CAGCTCCAAAGC	*KlALT1* His-tag, in bold restriction site for NdeI
X36	CCTTCTTTGACAAGTACCGTGAT TAG**CTCGAG**GCGCGC	*KlALT1* His-tag, in bold restriction site for XhoI
X37	TGACATGTTAGAGGAGATTCCT CGC	*LkALT1* Northern blot probe
X38	CCACCAACCCTGAAGAGATCGA A	*LkALT1* Northern blot probe
X39	TGCTTACTCTCAATCACAGGG	*KlALT1 Northern blot probe*
X40	CCTCAAGGTGCCATGTACCT	*KlALT1* Northern blot probe
X41	AAGCTCGTAGTTGAACTTTGGG TCTGG	*Lk18S* and *Kl18S* Northern blot probe
X42	CGGCACCTTACGAGAAATCAAA GTCTT	*Lk18S* and *Kl18S* Northern blot probe
