# Supplementary material for: Evolutionary Diversification of Alanine Transaminases in Yeast: Catabolic Specialization and Biosynthetic Redundancy
Source: Front Microbiol. 2017 Jun 26;8:1150. doi: 10.3389/fmicb.2017.01150 (PMC5483587; doi:10.3389/fmicb.2017.01150)
Supplement: Supplementary file 2 [file Table_2.PDF]

Evolutionary Diversification of Alanine Metabolism in Yeast: Catabolic Specialization and Biosynthetic Redundancy

Ximena Martínez de la Escalera-Fanjul, Carlos Campero-Basaldua, Maritrini Colón, James González, Dariel Márquez, and Alicia González[1]*

*Author for correspondence:
Alicia González
amanjarr@ifc.unam.mx

Table S2. Primers used for nucleosome scanning assays in *LkALT1* locus

Name	Sequence	Application
E1	Fw AAT CAT GAC CCG AGG TCT TAC<br>Rv AAA GTA CAC GCC ATT CAA GCA	-940 to -834
E2	Fw ACC ACA CTC ACT GAG CAA G<br>Rv TAG TGA TAA ACG TCT GGT ACA G	-890 to -789
E3	Fw TGC TTG AAT GGC GTG TAC TTT<br>Rv CAA GAA AGA AGC TGT TAG TGT CA	-854 to -755
E4	Fw CTG TAC CAG ACG TTT ATC ACT A<br>Rv TCT TGA GAA CAC TGG TTG CAC	-810 to -711
E5	Fw TGA CAC TAA CAG CTT CTT TCT TG<br>Rv CTG TCG GTA AGA ACG TTG TAT	-777 to -664
E6	Fw GTG CAA CCA GTG TTC TCA AGA<br>Rv TGG GTT TTC GTA GTT TTT AGT GC	-731 to -630
E7	Fw ATA CAA CGT TCT TAC CGA CAG<br>Rv GTT GTT GTT TTT TCA TTT TTC CAC G	-684 to -593
E8	Fw GCA CTA AAA ACT ACG AAA ACC CA<br>Rv ATG TAT CGC TCG AGC CAG T	-652 to -554

Name	Sequence	Application
E9	Fw CGT GGA AAA ATG AAA AAA CAA CAA C<br>Rv ACC TCT TTT CTC GGC TGT GT	-617 to -521
E10	Fw ACT GGC TCG AGC GAT ACA T<br>Rv GTA TAT GGG AGA AAG AGA AGC	-572 to -471
E11	Fw ACA CAG CCG AGA AAA GAG GT<br>Rv CTG TAT GAA AGT AGT AGA AGC G	-540 to -438
E12	Fw GCT TCT CTT TCT CCC ATA TAC<br>Rv CTC TAT ACG GCT CGT TCT GTA	-492 to -395
E13	Fw CGC TTC TAC TAC TTT CAT ACA G<br>Rv ACT GCC TTG CCG TTT TTC C	-459 to -361
E14	Fw TAC AGA ACG AGC CGT ATA GAG<br>Rv TGT TCA ATC AGC GAC GGC T	-415 to -312
E15	Fw GGA AAA ACG GCA AGG CAG T<br>Rv CTT CAA AGG GCG CCT ATT GTA	-379 to -280
E16	Fw AGC CGT CGC TGA TTG AAC A<br>Rv TGC CAA GAT TGT ACC AGT AGA A	-330 to -239
E17	Fw TAC AAT AGG CGC CCT TTG AAG<br>Rv GTA CTT GCC GGA CAA AAC CA	-300 to -201
E18	Fw TTC TAC TGG TAC AAT CTT GGC A<br>Rv AAT TGA AAC GAT TTT TGT CGA TGT T	-260 to -173
E19	Fw TGG TTT TGT CCG GCA AGT AC<br>Rv TAA CGG AAA GGA GGA GAA ATC T	-220 to -122
E20	Fw AAC ATC GAC AAA AAT CGT TTC AAT T<br>Rv CCG TGA AAA GGC GTG AAA AAT TT	-197 to -85
E21	Fw AGA TTT CTC CTC CTT TCC GTT A<br>Rv AAC GAA GGG GTG GGA CGT	-143 to -51

Name	Sequence	Application
E22	Fw AAA TTT TTC ACG CCT TTT CAC GG<br>Rv TGA CGA GAG TTG TTT GCG TGA	-107 to -11
E23	Fw ACG TCC CAC CCC TTC GTT<br>Rv GCA TTC TTA AGC TAG GAG TTC T	-68 to +40
E24	Fw TCA CGC AAA CAA CTC TCG TCA<br>Rv TAG CGA TAT TAC CAG CAG TAG	-31 to +70
E25	Fw AGA ACT CCT AGC TTA AGA ATG C<br>Rv TAT AGG GCA CAA AGT TCG ACG	+19 to +130
