# Supplementary material for: Evolutionary Diversification of Alanine Transaminases in Yeast: Catabolic Specialization and Biosynthetic Redundancy
Source: Front Microbiol. 2017 Jun 26;8:1150. doi: 10.3389/fmicb.2017.01150 (PMC5483587; doi:10.3389/fmicb.2017.01150)
Supplement: Supplementary file 3 [file Table_3.PDF]

Evolutionary Diversification of Alanine Metabolism in Yeast: Catabolic Specialization and Biosynthetic Redundancy

Ximena Martínez de la Escalera-Fanjul, Carlos Campero-Basaldua, Maritrini Colón, James González, Dariel Márquez, and Alicia González[1]*

*Author for correspondence:
Alicia González
amanjarr@ifc.unam.mx

Table S3. Primers used for nucleosome scanning assays in *KlALT1* locus

Name	Sequence	Application
F1	Fw TTC ACG ACA CTG TAT TAA GCG TA<br>Rv ATT TTG CGG GTA TCT TGT TGC AA	-903 to -802
F2	Fw CTT AAG GTG GAA AGT CGG AAG<br>Rv CTC GGT GTA GAT ACT ATT TAA CC	-860 to -769
F3	Fw TTG CAA CAA GAT ACC CGC AAA AT<br>Rv AGG ACT GAT AGA AAA GTG GAC A	-824 to -731
F4	Fw GGT TAA ATA GTA TCT ACA CCG AG<br>Rv AAA GTA AGC ATC AAC CGC CCA	-791 to -693
F5	Fw TGT CCA CTT TTC TAT CAG TCC T<br>Rv GGC ATT GCA CAT CAA GGG TT	-752 to -655
F6	Fw TGG GCG GTT GAT GCT TAC TTT T<br>Rv GCA AAA TAG TGC TGT AAG GAA AA	-713 to -620
F7	Fw AAC CCT TGA TGT GCA ATG CC<br>Rv GCT CAT CAT TTT GAA TAT AAC AAT TG	-674 to -584
F8	Fw TTT TCC TTA CAG CAC TAT TTT GC<br>Rv TGG GAC TGT GTT GAT CTG AAA T	-642 to -542
F9	Fw CAA TTG TTA TAT TCA AAA TGA TGA GC<br>Rv AGC CTT AAA CCC AAA AGA AAA ACT A	-612 to -504

Name	Sequence	Application
F10	Fw ATT TCA GAT CAA CAC AGT CCC A<br>Rv GTA ACC CTT TAT CTT TTT TTT TCC T	-563 to -461
F11	Fw TAG TTT TTC TTT TGG GTT TAA GGC T<br>Rv ATG GAG GCA AGG ATT CAA AGT AT	-528 to -428
F12	Fw AGG AAA AAA AAA GAT AAA GGG TTA C<br>Rv TAA TAG CGT AGT CCC GTC TTG A	-485 to -381
F13	Fw ATA CTT TGA ATC CTT GCC TCC AT<br>Rv ACA ACT GTA CGT AAC AAA TGA ACA	-450 to -347
F14	Fw TCA AGA CGG GAC TAC GCT ATT A<br>Rv CTG ATA ACG AAA AAC TAA AAA GTG	-402 to -293
F15	Fw TGT TCA TTT GTT ACG TAC AGT TGT<br>Rv AAA AGA TGG AAT GGC TGA CCA	-370 to -270
F16	Fw CAC TTT TTA GTT TTT CGT TAT CAG<br>Rv TCT TCT TTT TCT TTC AGT GTC AGA	-316 to -209
F17	Fw TGG TCA GCC ATT CCA TCT TTT<br>Rv AAT GTA TTC TCG ACC TAT AGG A	-290 to -179
F18	Fw TCT GAC ACT GAA AGA AAA AGA AGA<br>Rv AAA ATT CGC CCA ACT AAA TCT CGA	-232 to -126
F19	Fw TCC TAT AGG TCG AGA ATA CAT T<br>Rv ACT AAA CGA GTA ACA GGA AAA C	-200 to -101
F20	Fw TCG AGA TTT AGT TGG GCG AAT TTT<br>Rv AGC CAA ATT CCT CTC GAA GCT	-149 to -64
F21	Fw GTT TTC CTG TTA CTC GTT TAG T<br>Rv AGT GTT TGA TGG AAA GCT CTT AAC	-122 to -7
F22	Fw AGC TTC GAG AGG AAT TTG GCT<br>Rv AGT TAA CTC TGA CAG ACA ACA TCT	-84 to +22

Name	Sequence	Application
F23	Fw GTT AAG AGC TTT CCA TCA AAC ACT<br>Rv AAA GCT GGA GCA TTG ACT CTA	-30 to +71
F24	Fw AGA TGT TGT CTG TCA GAG TTA ACT<br>Rv TAA ACT TGT TAC AGA AGG CAT TGT	-2 to +102
F25	Fw TAG AGT CAA TGC TCC AGC TTT<br>Rv TAA CAT CAT CTA GCG AAA GCT TTG	+50 to +163
