# Supplementary material for: Evolutionary Diversification of Alanine Transaminases in Yeast: Catabolic Specialization and Biosynthetic Redundancy
Source: Front Microbiol. 2017 Jun 26;8:1150. doi: 10.3389/fmicb.2017.01150 (PMC5483587; doi:10.3389/fmicb.2017.01150)
Supplement: Supplementary file 4 [file Image_1.PDF]

Evolutionary Diversification of Alanine Metabolism in Yeast: Catabolic Specialization and Biosynthetic Redundancy

Ximena Martínez de la Escalera-Fanjul, Carlos Campero-Basaldua, Maritrini Colón, James González, Dariel Márquez, and Alicia González[1]*

*Author for correspondence:
Alicia González
amanjarr@ifc.unam.mx

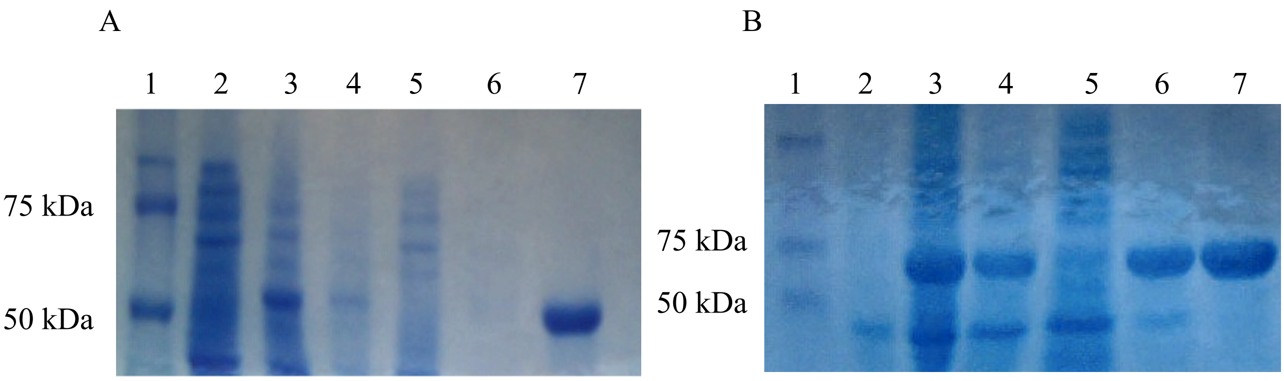

Figure S1. ***Lk*At1 and *Kl*Alt1 purification.** Recombinant enzymes from *L. kluyveri* and *K. lactis* were produced in *E. coli*. A) *Lk*Alt1 (61.16 kDa): Lane 1, Ladder; Lane 2, non induced cellular extract; Lane 3, induced cellular extract; Lane 4, soluble fraction; Lane 5, not attached fraction; Lane 6, washing 30 mM imidazol; Lane 7, elution 200 mM imidazol. B) *Kl*Alt1 (59.23 kD): Lane 1, Ladder; Lane 2, non induced cellular extract; Lane 3, induced cellular extract; Lane 4, soluble fraction; Lane 5, not attached fraction; Lane 6, washing 30 mM imidazol; Lane 7, elution 200 mM imidazol.
