# Supplementary material for: Evolutionary Diversification of Alanine Transaminases in Yeast: Catabolic Specialization and Biosynthetic Redundancy
Source: Front Microbiol. 2017 Jun 26;8:1150. doi: 10.3389/fmicb.2017.01150 (PMC5483587; doi:10.3389/fmicb.2017.01150)
Supplement: Supplementary file 5 [file Image_2.PDF]

Evolutionary Diversification of Alanine Metabolism in Yeast: Catabolic Specialization and Biosynthetic Redundancy

Ximena Martínez de la Escalera-Fanjul, Carlos Campero-Basaldua, Maritrini Colón, James González, Dariel Márquez, and Alicia González[1]*

*Author for correspondence:
Alicia González
amanjarr@ifc.unam.mx

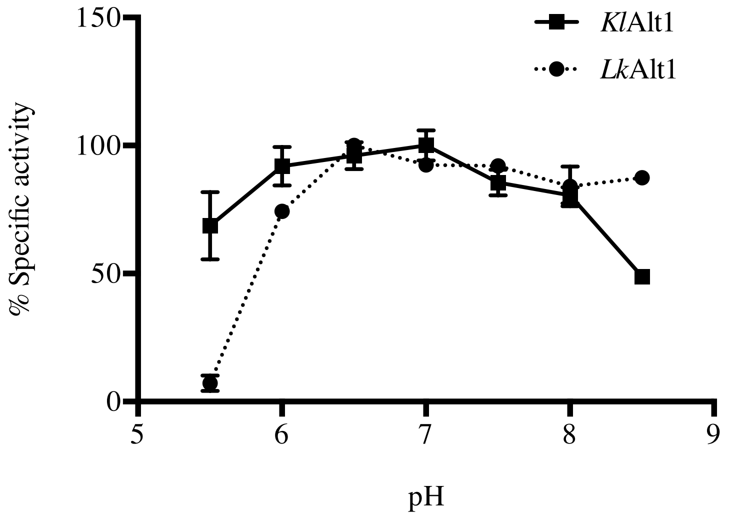

Figure S2. ***Lk*At1 and *Kl*Alt1 pH response.** After heterologous expression and purification, *Lk*At1 and *Kl*Alt1 specific activity was measured at different pH. results are presented as % of the specific activity. Values are presented as means ± SD from three independent experiments.
